# Safety and efficacy of C1-inhibitor in traumatic brain injury (CIAO@TBI): study protocol for a randomized, placebo-controlled, multi-center trial

**DOI:** 10.1186/s13063-021-05833-1

**Published:** 2021-12-04

**Authors:** Inge A. M. van Erp, Thomas A. van Essen, Kees Fluiter, Erik van Zwet, Peter van Vliet, Frank Baas, Iain Haitsma, Dagmar Verbaan, Bert Coert, Godard C. W. de Ruiter, Wouter A. Moojen, Mathieu van der Jagt, Wilco C. Peul

**Affiliations:** 1 University Neurosurgical Center Holland, Leiden University Medical Center, Haaglanden Medical Center and Haga Teaching Hospital, Albinusdreef 2, J-11-R-83, 2333 ZA Leiden, Hague The Netherlands; 2 Department of Clinical Genetics, Leiden University Medical Center, Leiden, The Netherlands; 3 Department of Biomedical Data Science, Leiden University Medical Center, Leiden, The Netherlands; 4 Department of Intensive Care, Haaglanden Medical Center, The Hague, The Netherlands; 5 Department of Neurosurgery, Erasmus MC – University Medical Center, Rotterdam, The Netherlands; 6 Neurosurgical Center Amsterdam, Amsterdam University Medical Center, Amsterdam, The Netherlands; 7 Department of Intensive Care Adults, Erasmus MC – University Medical Center, Rotterdam, The Netherlands

**Keywords:** Traumatic brain injury, C1-inhibitor, Neuroinflammation, Randomized controlled trials

## Abstract

**Background:**

Traumatic brain injury (TBI) is a major cause of death and disability across all ages. After the primary impact, the pathophysiologic process of secondary brain injury consists of a neuroinflammation response that critically leads to irreversible brain damage in the first days after the trauma. A key catalyst in this inflammatory process is the complement system. Inhibiting the complement system could therefore be a therapeutic target in TBI.

**Objective:**

To study the safety and efficacy of C1-inhibitor (C1-INH) compared to placebo in patients with TBI. By temporarily blocking the complement system, we hypothesize a decrease in the posttraumatic neuroinflammatory response resulting in a less unfavorable clinical outcome for TBI patients.

**Methods:**

CIAO@TBI is a multicenter, randomized, blinded, phase II placebo-controlled trial. Adult TBI patients with GCS < 13 requiring intracranial pressure (ICP) monitoring will be randomized, using block randomization, within 12 h after trauma to one dose 6000 IU C1-INH or placebo. A total of 106 patients will be included, and follow-up will occur up to 12 months. The primary endpoints are (1) Therapy Intensity Level (TIL) Scale, (2) Glasgow Outcome Scale-Extended (GOSE) at 6 months, and (3) complication rate during hospitalization. Outcomes will be determined by a trial nurse blinded for the treatment allocation. Analyses will be conducted in an intention-to-treat analysis.

**Discussion:**

We expect that C1-INH administration will be safe and potentially effective to improve clinical outcomes by reducing neuroinflammation in TBI patients.

**Trial registration:**

ClinicalTrials.gov NCT04489160. Registered on 27 July 2020. EudraCT 2020-000140-58

**Supplementary Information:**

The online version contains supplementary material available at 10.1186/s13063-021-05833-1.

## Background

Traumatic brain injury (TBI) is a major cause of death and disability around the world. In Europe, over one million traumatic brain injury (TBI) patients are admitted to the hospital yearly, of whom 75,000 people die [1, 2]. This debilitating morbidity leads to enormous societal costs [3]. Therapies and guidelines that have been demonstrated to improve the outcomes after TBI are still limited, especially in the management of severe TBI (s-TBI), defined by Glasgow Coma Scale (GCS) 3–8 [4]. Patients with s-TBI have a high mortality rate, estimated at 30–40% in observational studies [1]. Survivors of s-TBI experience a substantial burden of physical, psychiatric, emotional, and cognitive disabilities that disrupt their lives, their proxy family members, and their social surroundings. Adequate treatment strategies are therefore pivotal. TBI comprises a dynamic pathophysiology that evolves over time, consisting of primary injury after the traumatic hit, followed by systemic disorders which lead to secondary injury [5]. The overshooting inflammatory response and delayed formation of brain edema, mostly seen around the third day after trauma, are characteristics of secondary brain injury, which are in turn related to a complicated clinical course, delayed recovery, late morbidity, and mortality in TBI [6–8].

The complement system forms the first line of defense against microorganisms and is critical in sensing tissue damage. Complement activation can be mediated by three distinct pathways: the classical pathway, the lectin pathway, and the alternative pathway [9]. Multiple experimental studies have identified a pathophysiologic role of the complement system in contributing to posttraumatic neuro-inflammation, disruption of the blood-brain barrier, secondary neuronal damage, and neuronal cell death after TBI [10–14]. Activation of the complement system in s-TBI results in a cascade of events including increased vascular permeability and activation of microglia and astrocytes, ultimately resulting in inflammatory reactions in and around contusion areas [15, 16]. In addition, it triggers a sustained degenerative mechanism of reduced dendritic and synaptic density and inhibited neuroblast migration several weeks after TBI in animal models [17]. In severe TBI patients, elevated complement factors have been found in the serum [18, 19] and in the ventricular cerebrospinal fluid (CSF) directly after initial trauma [20, 21].

**Fig. 1:**
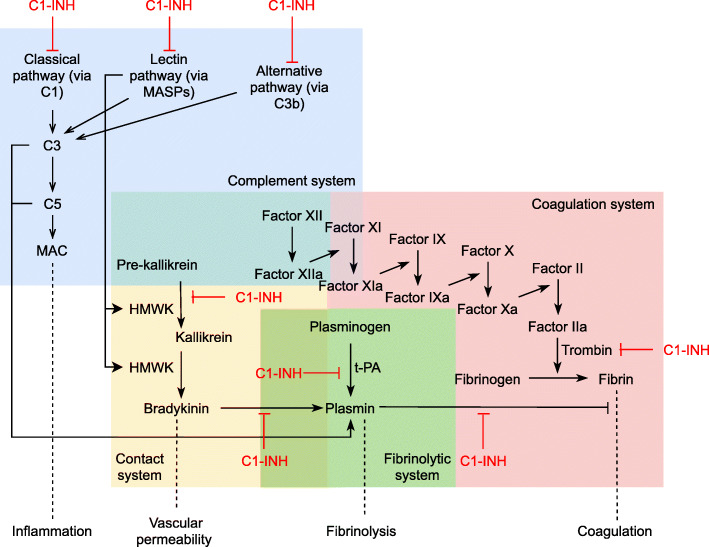
Contact, complement, coagulation, and fibrinolytic systems and targets of C1-INH. *Abbreviations*: MAC, membrane attack complex; HMWK, high-molecular-weight kininogen; C1-INH, C1-inhibitor; t-PA, tissue plasminogen activator; MASPs, mannose-associated serine protease. *Explanation*: C1-INH is directed at all three pathways of the complement system, but has also an effect on the contact, fibrinolytic, and coagulation system

Complement inhibition is therefore considered to be a potentially important target of TBI treatment. Inhibitors of C3 or C5 convertases, membrane attack complex (MAC) formation, and C1-inhibitor (C1-INH) have been identified to prevent secondary neurologic damage and improve neurologic performance in mice by reducing microglia activation, apoptosis, and axonal loss [22–26]. C1-INH is an inhibitor of the classical, lectin, and alternative pathway, which additionally interact with the contact and coagulation system, and is currently indicated for the treatment of hereditary angioedema (HAE) [27–29] (Fig. 1). Because of its favorable safety profile, it has been used to treat other inflammatory diseases, such as sepsis or ischemic reperfusion injury [30, 31].

High-quality preclinical evidence suggests a significant role of the complement system in the pathophysiology of neuroinflammation. As complement inhibition is potentially an important aspect of TBI treatment to attenuate neuroinflammation, the CIAO@TBI multicenter, randomized, blinded, placebo-controlled trial aims to assess the safety and efficacy of C1-INH in TBI patients. It is hypothesized that C1-INH can be beneficial in the treatment of s-TBI by decreasing the detrimental neuroinflammation and preventing secondary brain injury to ensure a more favorable functional outcome and a less hampered quality of life for the future TBI patients.

## Methods

### Trial design

Complement inhibition: Attacking Overshooting inflammation @fter Traumatic Brain Injury (CIAO@TBI) is a multicenter, randomized, blinded, placebo-controlled, phase II trial in patients with TBI. The protocol is designed in accordance with the Standard Protocol Items: Recommendation for Interventional Trials (SPIRIT) 2013 Checklist (Additional file 1) [32]. Patients will be recruited in three-level one trauma centers, including the coordinating neurotrauma research unit of University Neurosurgical Center Holland seated at Leiden University Medical Center (LUMC) and Haaglanden Medical Center (HMC) in close collaboration with Erasmus Medical Center Rotterdam (EMC) and Amsterdam University Medical Centers, location AMC. Patients will be randomized at the ICU in the first 12 h after trauma to receive (1) one dose of C1-INH 6000 IU intravenously (IV) or (2) one placebo injection with 0.9% saline IV.

### Objectives

The main objective of the CIAO@TBI trial is to determine the safety and efficacy of C1-INH in patients with s-TBI. The primary endpoints are (1) efficacy: intracranial pressure (ICP) directed Therapy Intensity Level (TIL), GOSE at 6 months after discharge, and (2) safety: complication rate during hospitalization.

### Trial population and eligibility

All TBI patients presenting to participating hospitals are potentially eligible for this trial. The inclusion criteria are (1) age > 18 years, (2) clinical diagnosis of TBI with GCS < 13 on admission (with visible intracranial pathology on CT), and (3) invasive intracranial pressure monitoring by intra-parenchymatous transducer or ventricular catheter placement and management of increased ICP for at least 24 h. The exclusion criteria are (1) a clear, non-traumatic cause of low GCS on admission (e.g., toxic or cardiac cause); (2) not expected to survive more than 24 h after admission with the decision to abstain from aggressive treatment and induce palliative care; (3) brain death on arrival in the participating centers; (4) severe pre-trauma disability, defined as being dependent on other people; (5) known prior history of sensitivity to blood products or C1-INH; (6) patients with a history of hereditary angioedema (if known at admission); (7) patients with a history of thrombosis (deep vein thrombosis, cerebral venous sinus thrombosis, or pulmonary embolism) (if known at admission); and (8) patients that are pregnant (if known at admission).

### Randomization

Patients in the trial are allocated to treatment with C1-INH or placebo in a 1:1 ratio, via a confidential interactive web-based algorithm software (Castor EDC, Ciwit B.V., Amsterdam, The Netherlands) using permuted block sized with stratification by study center. Randomization will be performed by the treating neurosurgeon, neurosurgical resident, or research physician after obtaining informed/deferred consent. An automatically generated email will be sent to the pharmacy with information regarding treatment allocation after randomization through Castor EDC.

### Trial interventions

**Fig. 2:**
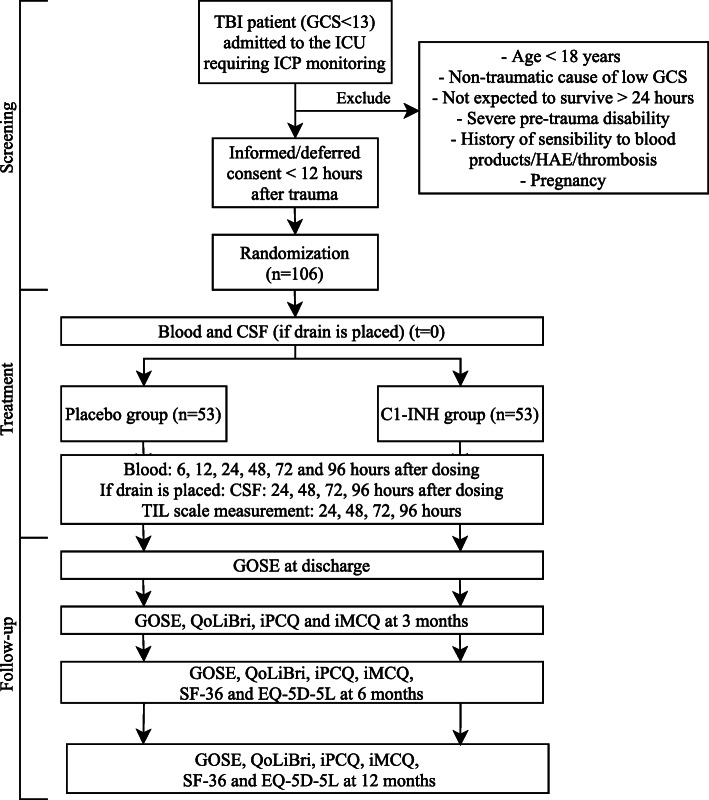
CIAO@TBI study design flowchart. *Abbreviations*: TBI, traumatic brain injury; GCS, Glasgow Coma Scale; HAE, hereditary angio-edema; CSF, cerebrospinal fluid; TIL, therapy intensity level; GOSE, Glasgow Outcome Scale Extended; QoLiBri, Quality of Life after Brain Injury Scale; iPCQ, IMTA Productivity Cost Questionnaire; iMCQ,: IMTA Medical Consumption Questionnaire; SF-36, Short-Form 36; EQ-5D-5L, 5 Level EuroQoL 5-Dimensional Questionnaire

The intervention is the IV administration of C1-INH compared to a placebo (0.9% saline). A single dose of 6000 IU will be administered within 12 h after trauma. Blood samples (2 EDTA, 1 serum, and 1 citrate tube) will be drawn from both patient groups before administration and at 6, 12, 24, 48, 72, and 96 h after administration of C1-INH or placebo in the ICU. Moreover, if an external venticular drain (EVD) is placed as part of standard care to reduce CSF pressure, additional liquor samples will be drawn before administration and 24, 48, 72, and 96 h after administration of C1-INH or placebo in the ICU. All samples will be centrifuged and aliquoted in 500 μl samples as quickly as possible and afterwards stored at − 80 °C. Other interventions are unaltered from local treatment protocols and will include standard of care in both groups. This might include concomitant therapies, such as a decompressive craniectomy. During hospitalization, patients are closely monitored for (serious) adverse events (SAEs). Moreover, health-related questionnaires will be filled out by the patients or proxies at discharge and at 3 months, 6 months, and 12 months after head trauma. A flowchart of the study design is shown in Fig. 2. All study procedures will be performed according to the designated study operating procedures (SOPs).

#### Investigational medicinal product

Cinryze (Takeda Development Center Americas, Inc., Lexington, MA) is a nano-filtered C1-inhibitor product, purified from human plasma for fractionation. It is provided as a freeze-dried powder and is reconstituted with 5 ml water at the site by a trained unblinded person. The final concentration of the drug substance is 100 IU/ml, and a total of twelve vials are used for each patient (6000 IU). The total of 60 ml C1-INH will be divided over two syringes and will be infused IV over 1 h. If the patient is randomized for the placebo group, the equivalent amount (60 ml) 0.9% saline will be dosed IV over 1 h.

#### Justification of route of administration and dosage

Administration of the study intervention via the intravenous route is required as subcutaneous dosing yields lower bioavailability and results in a higher incidence of injection site reactions [33]. Moreover, this route is feasible in critically ill patients. The dose of C1-INH or placebo equivalent to be used is based on pharmacokinetics/pharmacodynamics, efficacy, and safety. The 1000/1500-IU dose as prescribed for HAE is enough to inhibit kallikrein-induced bradykinin generation, but not enough to inhibit complement activation (Fig. 1). Safety studies showed that the no observed adverse effect level (NOEL) is 400 U/kg, and 1 unit is necessary to prevent complement activation in 1 ml blood [34]. The efficacy and safety of 6000 IU C1 esterase inhibitor treatment have not yet been tested in TBI patients. Nevertheless, this high dose has been used in critically ill patients before. In a trial in severe sepsis patients, a regime of 6000 IU followed by 3000, 2000, and 1000 IU was used to efficiently block complement activation [30]. This dosage was well tolerated in these critically ill patients, with no drug-related adverse events. Currently, two other RCTs, one evaluating asthma treatment and one focusing on anemia, are recruiting patients with the same dose of 6000 IU Cinryze. Furthermore, other studies have already shown safety of Cinryze at doses of 12,000, 15,000, and 19000 units per patient [31, 35, 36]. A minimal of 6000 IU (which is equivalent to 85 IU/kg for a person weighing 70 kg) is justified to achieve treatment effect without major complications to be expected.

The time window of administration of the investigational product within 12 h after trauma is defined based on the therapeutic window of C1-INH together with the required time to stabilize the critically ill patient and provide the required first urgent interventions. Nevertheless, animal models show that the closer the administration is to the actual trauma, effects are more beneficial, emphasizing the need for early as possible intervention.

### Treatment blinding

Patients, care providers, site investigators, research coordinators, and statisticians will be blinded to treatment allocation. C1-INH and placebo are prepared by an unblinded staff member at each center to provide a ready-to-use solution. This will be a pharmacist or nurse from an independent department, depending on the local site organization, and this person will not be involved in the care of the trial patients and will not discuss trial drug treatment with research staff or other members of the ICU. Preparation and labeling will be done according to the Good Manufacturing Practice Guidelines. The unblinded staff member will provide the C1-INH or placebo in two masked syringes, labeled with a randomization number and infusion rate, to the blinded treatment team. Both the reconstituted C1-INH salutation as the placebo solution are colorless, and provided in identical syringes, therefore maintaining blinding for the treatment team. The trained ICU nurse will infuse the C1-INH or placebo. For safety reasons, emergency unblinding can be performed through Castor EDC.

### Primary endpoints

#### Efficacy: Therapy Intensity Level (TIL) Scale

To measure the direct effect on the mechanism of action of C1-INH, our primary endpoint focuses on a decrease in ICP. ICP has been used as a surrogate endpoint for inflammation and testing of neuroprotective agents in many prematurely halted or failed clinical trials [37]. This might be due to the fact that ICP is an early surrogate marker during TBI but confounded by the modern neuro-ICU practices through escalating interventions (such as decompressive craniectomies (DC)) to immediately mitigate surges in ICP resulting in reduction of its sensitivity, as representing “severity of disease.” Therefore, this trial will focus on the intensity of ICP-targeted therapy based on the Therapy Intensity Level (TIL) Scale [38, 39]. The TIL Scale is designed to integrate all known and relevant ICP directed treatments into a single-summary score and was developed as part of the Interagency Common Data Elements scheme [38]. Since its introduction, the novel TIL Scale has been widely used in neurotrauma research, with excellent inter- and intra-rater reliability with minimal measurement errors [39]. The TIL Scale includes eight ICP treatment modalities each with a certain numerical score. Added together, TIL ranges between 0 and 38 points with a right-skewed distribution. Specifically, TBI patients admitted to the ICU have an average TIL score of 8.2 (SD 3.2), while general trauma patients on the ICU have a score of 2.2 (SD 0.9) [39]. The daily score will be calculated based on the highest score in each item per day (TIL24), to provide a metric on the maximal therapeutic intensity for ICP management for that day. The TIL score will be based on the highest TIL24 during the days an ICP monitor is in place (TILmax) and will be scored by a trained research team, and according to an SOP, thereby optimizing inter- and interrater reliability.

#### Efficacy: GOSE at 6 months

The co-primary efficacy endpoint will be the Extended Glasgow Outcome Scale (GOSE) at 6 months after trauma [40]. The GOSE [41], derived from its precursor the GOS [42], defines disability on an 8-point scale and incorporates emotional and cognitive disturbances affecting disability. A score of 5 or more indicates functional independence. The GOSE is designed as a structured interview and can also be applied through telephone [43] and e-mail or postal [44]. Two research nurses or researchers will independently grade the outcomes based on the GOSE in each patient according to the standardized approach. Disagreements will be resolved by consensus between them or by consultation of a third, independent investigator.

#### Safety: complication rate during hospitalization

A complication rate during hospitalization will be calculated to determine the safety of C1-INH. This rate includes serious adverse events possibly related to study medication. This includes venous thromboembolic events (deep venous thrombosis and pulmonary embolism), myocardial infarction, ischemic stroke, meningitis, pneumonia, and sepsis. Patients will be assessed daily by a blinded physician/nurse for these complications. Vital signs will be monitored closely and potential adverse reactions to the experimental treatment will be picked up immediately at the ICU.

### Secondary endpoints

Secondary endpoints include all-cause mortality rate during the study period (proportion), ICP burden (pressure and time dose (PTD) calculated as the area under the curve above ICP threshold of 20 mmHg) during ICP monitoring, occurrence of CT midline shift during hospitalization (proportion), ICU and hospital length of stay (median), and hospital discharge location (proportion). Additionally, the GOSE will be assessed at discharge and 3 and 12 months follow-up. Quality of life will be assessed using the Quality of Life after Brain Injury Scale (QoLiBri) [45, 46]. The QoLiBri is the first TBI disease-specific quality of life outcome tool that is cross-culturally developed and validated in large populations. This questionnaire consists of 37 items designed to assess the health-related quality of life (HRQol) after TBI, and the median score will be compared at 3, 6, and 12 months after trauma. To assess health and well-being, the Short Form (SF)-36 [47] and 5-Level EuroQol 5-Dimensional Questionnaire (EQ-5D-5L) will be assessed [48]. SF-36 is a subjective measure of health and well-being, and the median score will be compared at 6 and 12 months after head trauma. The EQ-5D-5L is a 5-dimensional generic instrument assessing health-related quality of life and health status and generates an index of health. This questionnaire will be filled out by patients at 6 and 12 months follow-up, and the median score will be compared between the groups. The cost-effectiveness of the C1-INH will be analyzed with the median costs per quality-of-life year (QALY), based on the iMTA Productivity Cost Questionnaire (iPCQ) and iMTA Medical Consumption Questionnaire (iMCQ) at 3, 6, and 12 month follow-up [49]. Blood and CSF samples will be used (up to 96 h after C1-INH administration) to measure complement activation in an exploratory fashion, using WIESLAB, C4b/C, C3b/C, and C5b-9 ELISA assays. Neurological damage will be analyzed with glial fibrillary acidic protein (GFAP) and ubiquitin C-terminal hydrolase-L1 (UCHL-1) biomarkers. To explore the interactions of the complement system with the coagulation cascade, routinely, PT, aPPT, PLT, d-dimer, and fibrinogen tests will be performed and compared between the treatment groups. The Disseminated Intravascular Coagulation (DIC) Score will be calculated for all patients as proposed by the Scientific and Standardisation Committee of the International Society on Thrombosis and Haemostasis [50]. Lastly, general inflammatory markers, TNF-alpha, and interleukins will be analyzed to explore the effect of C1-INH on neuroinflammation.

### Sample size

To detect a between-group difference in the TIL Scale of 2.2, with 90% power and 0.05 (two-sided) significance level, a total of 106 patients are required. Calculations were based on the anticipated mean of 8.2 and standard deviation 3.2 of the novel TIL in TBI patients admitted to the ICU [38]. The patient group treated with C1-INH is estimated to have an average TIL of 6 [39, 51]. Both treatment groups will include 53 patients to power the trial and to account for withdrawal and loss to follow-up (approximately 10%). With this sample size, a difference of 2.2 on the TIL scale can be detected, which is clinically relevant as it corresponds to one medium level of intervention (such as CSF drainage or mannitol treatment) less to control ICP indicating a significant treatment effect. As no minimal clinically important difference (MCID) for the TIL scale is defined in the literature, we will adhere to this shift of 2.2 points on the right-skewed scale.

### Statistical analysis

The trial profile will be summarized using a Consolidated Standards of Reporting Trials (CONSORT) flow diagram, including the reasons for non-eligibility and non-enrollment [52]. The data will be analyzed on an intention-to-treat basis with all randomized patients included in the analysis. Baseline variables will be summarized using descriptive statistics. In line with the IMPACT recommendations, the investigators plan to analyze the primary outcome TIL by using a linear model with covariate adjustment for age, GCS, and pupillary reactivity to adjust for baseline imbalances and to optimize statistical efficiency [53]. TIL will be analyzed as the TILmax for each patient, and the median for each group will be calculated. The treatment effect estimated will be based on the adjusted mean differences including 95% confidence intervals. To maintain the 5% familywise error rate, while still comparing the multiple clinically relevant endpoints, we will use a serial gatekeeping approach for statistical testing [54]. Analysis of GOSE at 6 months, as an ordinal outcome, will be done only if a significant difference in TIL is found. The co-primary endpoint GOSE will not primarily be used to declare the study success. For the analysis of GOSE at 6 months, covariate adjustment for the strongest predictors of outcome in TBI will be performed as mentioned above. The effect estimate will be an adjusted proportional odds ratio with 95% confidence interval for the shift in the direction of a better outcome on the GOSE. The ordinal regression model assumes that the odds ratio for each potential cut of the GOSE is constant no matter which cutoff point is taken (proportional odds assumption). Although the common odds ratio is formally only valid if the proportional odds assumption is met, the common odds ratio can be interpreted as a summary measure of treatment effect, even if the odds ratios differ by cutoff [55, 56]. In this study, the resulting single, common odds ratio can be interpreted as the average shift over the GOSE scale at 6 months caused by C1-INH compared to placebo [56].

Secondary analyses will be compared between the treatment groups using unadjusted and adjusted (ordinal) logistic and linear regression. Time-to-event analyses will be undertaken using Kaplan-Meier curves, as well as Cox proportional hazards regression models.

Subgroup analyses on primary outcomes will be performed in the following subgroups: age (< 65 or ≥ 65 years), GCS on admission (moderate and severe TBI), and ICP-directed treatments (high intensity vs. low intensity, secondary DC vs. no secondary DC), and will be presented in forest plots.

#### Cost-effectiveness analysis

A trial-based cost-utility analysis (CUA: cost-per-QALY) will be performed from a societal perspective, extrapolated to a life-time horizon. Quality of life and cost questionnaires will be filled out by the patients at discharge and at 3, 6, and 12 months after trauma. Average discounted costs and QALYs will be compared according to intention-to-treat, using net-benefit analysis, and using multiple imputations to account for missing data. Estimated societal costs will include hospital costs (estimated from study registrations), other healthcare (using iMCQ questionnaire), and productivity (using iPCQ questionnaire). Healthcare will be valued using Dutch reference prices, including time and travel costs. Productivity losses will be valued using the friction-cost method (and the human-capital method as sensitivity analysis). A state transition model will be used to extrapolate survival and other outcomes beyond the trial duration, to a life-time horizon. QALYs will be calculated using the Dutch tariff for the EQ-5D-5L measures.

#### Missing data

Missing baseline data will be multiply imputed (*n* = 5), assuming data to be missing at random.

All analyses will be performed using the R statistical software (latest available version) with the required add-on R packages.

### Data monitoring

#### Data collection and protection

**Fig. 3:**
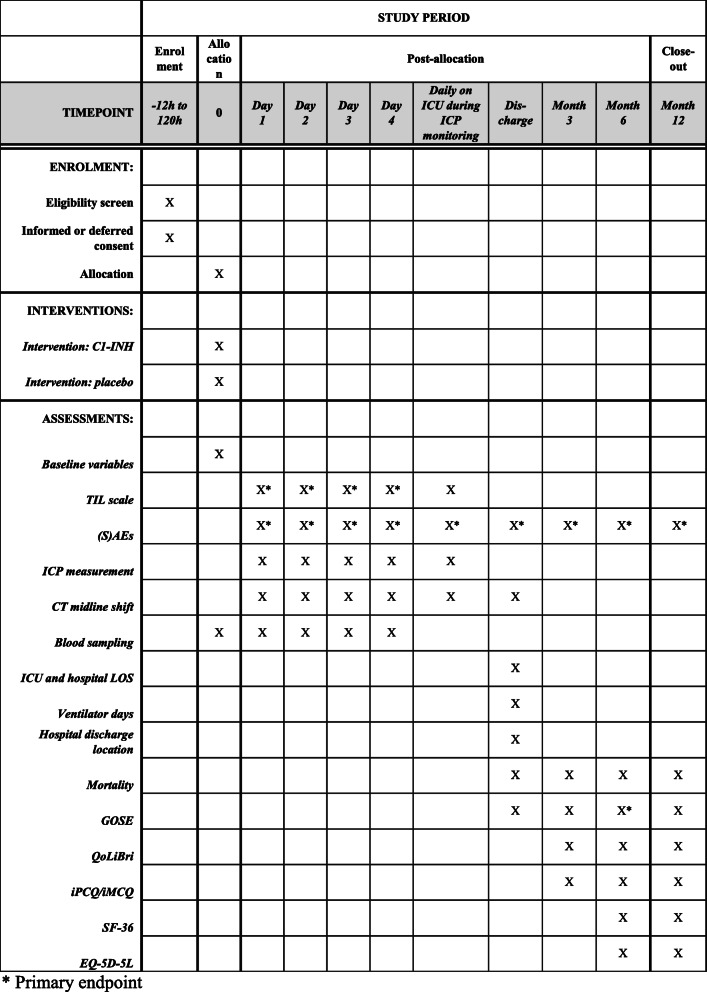
SPIRIT diagram of the recommended content for the schedule of enrollment, interventions, and assessments. *Abbreviations*: C1-INH, complement 1-inhibitor; TIL, therapy intensity level; (S)AEs, (serious) adverse events; ICP, intracranial pressure; CT, computed tomography; ICU, intensive care unit; LOS, length of stay; GOSE, Glasgow Outcome Scale Extended; QoLiBri, Quality of Life after Brain Injury Scale; iPCQ, iMTA Productivity Cost Questionnaire; iMCQ, iMTA Medical Cost Questionnaire; SF-36, Short-Form 36; EQ-5D-5L, 5 Level EuroQoL 5-Dimensional Questionnaire

Data will be collected, by dedicated research nurses and investigators, using Castor EDC, a web-based secured data capture platform. All subject data will be pseudonymized by assigning study numbers to each subject. The key to these study numbers is only available to the study team and an independent monitor from the LUMC. Data will be collected and stored for a period of 25 years. Biological samples will only be stored for the purpose of additional research if the patient has given consent. A SPIRIT diagram of the recommended content for the schedule of enrollment, intervention and data collection is included in Fig. 3.

#### Data and safety monitoring

An independent Data Safety Monitoring Board (DSMB) will monitor the safety of the trial with access to unblinded data regarding (S)AEs, suspected unexpected serious adverse events (SUSARs), and mortality. No interim analysis on the efficacy will be performed for this trial due to the relatively small sample size and the nature of a phase II trial focusing on safety. The DSMB will meet dependent on inclusion rate, with scheduled meetings after inclusion of 10, 30, 60, and 106 patients. The DSMB can advise the principal investigator to prematurely terminate the study if there is evidence of an unacceptable risk for trial subjects based on SAE reporting, outcome, and case fatality. Monitoring and auditing in all sites will be executed by monitors according to the pre-defined monitor plan. This will be independent from investigators and the sponsor, and they will ensure that adequate enrollment is met.

#### Safety and adverse event analysis

Safety analyses, as part of the primary endpoint, will be based on the safety set, consisting of the pre-defined SAEs, and will comprise standard descriptive methods. Changes from baseline will be summarized using standard statistical characteristics and shift tables. Details of the pre-defined SAEs, signs, and symptoms will be collected including details of onset, resolution, frequency, severity (mild, moderate or severe), seriousness, relationship to the drug, effect on the study drug, treatment administered, and outcome. Moreover, suspected unexpected serious adverse reactions (SUSARs) will be registered.

### Patient and public involvement

Patients and their caregivers have been involved in the research design, including selection of outcome measures and feasibility of the trial interventions and follow-up. The patient panel and their caregivers will be informed about the developments of the study and will be invited to participate in research meetings and discussions.

### Ethics

The study protocol was designed in accordance with the ethical principles in the Declaration of Helsinki and the regional regulations, and the principles of Good Clinical Practice. Participants will be assessed for eligibility by the neurosurgical resident or research physician when an ICP monitor is placed within 12 h from trauma. Within this time frame, informed consent must also be obtained. Ideally, the treating physician will obtain informed consent from the patient. As the enrolled subjects will lack capacity to consent due to their level of consciousness at presentation, informed consent can be obtained within 12 h after trauma by a legally authorized representative of the patient. As TBI mostly occurs outside the domestic situation, family members are rarely available during the first hours after trauma [57]. Therefore, the treating physician will take responsibility for including the patient using “deferred proxy consent” if the following condition are met and documented: (1) the patients is in a potentially life-threatening situation and (2) the patient meets the eligibility criteria for trial entry. As there is a potential benefit with the administration of C1-INH and an urgent therapeutic timeframe of 12 h, deferred consent is justified in these patients [58–60]. Following enrollment via deferred consent, informed consent should still be obtained by a legal representative within 7 days after admission. Furthermore, the patient must agree to further participate in the study when mental capacity is regained. When consent for study continuation is provided, already collected data can be used. When study continuation is refused, already collected data can still be used when patients and/or proxies do not use their right to refuse this. Patients or proxies should always be informed about their right to refuse the use of obtained data. A team of dedicated research nurses and physicians are trained to perform the specific informed consent and randomization procedures. Moreover, a 24/7 study consultation telephone number can be reached to help with problems or questions during the study.

### Reporting and dissemination

Reporting of our study will follow the CONSORT guidelines [52]. The results of this study will be presented at national and international scientific conferences and will be published in peer-reviewed journals without any publication restrictions. All authors who fulfil the authorship criteria will be included in future publications. There is no intended use of professional medical writers. Furthermore, the results of the current trial will inform a phase III clinical trial with a large and pragmatic enrollment rate powered solely on efficacy regarding clinical outcome improvement.

### Trial status

The trial will be conducted according to the protocol, version 7.0 (February 2021). Recruitment of subjects is intended to start February 2021 and target recruitment should be achieved by February 2023, making final outcomes available by the end of 2023.

#### Data Safety Monitoring Board

Prof. Dr. J van der Naalt, neurologist (University Medical Center Groningen, UMCG); Dr. M. Aries, neuro-intensivist (Maastricht University Medical Center, MUMC); and Prof. E. Steyerberg, statistician (Leiden University Medical Center, LUMC)

## Supplementary Information

**Additional file 1.** Standard Protocol Items: Recommendations for Interventional Trials (SPIRIT) 2013 checklist.

**Additional file 2.** Ethical approval document.

**Additional file 3.** Copy of the original funding documentation.

**Additional file 4.** Informed consent form.

## Data Availability

Final study datasets will be stored locally and securely at Leiden University Medical Center for long-term storage and access. These datasets will be available for researchers actively contributing to statistical analyses and publications. Data will be available upon request. Requests can be made with the corresponding author.

## Abbreviations

C1-INH: Complement 1-inhibitor
DSMB: Data Safety Monitoring Board
EQ-5D-5L: 5 Level EuroQoL 5-Dimensional Questionnaire
GCS: Glasgow Coma Scale
GOSE: Glasgow Outcome Scale Extended
ICP: Intracranial pressure
iPCQ: IMTA Productivity Cost Questionnaire
iMCQ: IMTA Medical Consumption Questionnaire
MAC: Membrane attack complex
PI: Principal investigator
SAE: Serious adverse event
SF-36: Short-Form 36
TBI: Traumatic brain injury
TIL: Therapy Intensity Level
QoLiBri: Quality of Life after Brain Injury Scale
