# Supplementary material for: Safety and efficacy of C1-inhibitor in traumatic brain injury (CIAO@TBI): study protocol for a randomized, placebo-controlled, multi-center trial
Source: Trials. 2021 Dec 4;22:874. doi: 10.1186/s13063-021-05833-1 (PMC8642972; doi:10.1186/s13063-021-05833-1)
Supplement: Supplementary file 2 — Additional file 2. Ethical approval document. [file 13063_2021_5833_MOESM2_ESM.pdf]

Medisch-Ethische Toetsingscommissie
Leiden | Den Haag | Delft

commissie METC-LDD
postzone P5-P
Mw. P.A. Visser

telefoon (071) 52 63241 of (071) 5266963
e-mail metc-ldd@lumc.nl
onze referentie **P20.021**/PV/pv
uw referentie
datum 12 april 2021
onderwerp **Statement on approval NL72551.058.20**

aan Prof. dr. W.C. Peul
Ms I. van Erp
LUMC

afdeling Neurochirurgie
postzone J11-R

To whom it may concern

On behalf ot the accredited medical research ethics committee (MREC) Leiden Den Haag Delft, I hereby declare that the committee reveiwed and approved research protocol "CIAO@TBI: Complement Inhibition: Attacking the Overshooting Inflammation @fter Traumatic Brain Injury (A phase II trial on the safety and efficacy of C1 esterase inhibitor Cinryze for the acute management of severe traumatic brain injury)", national registration number NL72551.058.20, MREC registration number P20.021.

Approval was given on 23 July 2020 and subsequent amendments were approved by the Committee on 19 November 2020, and 6 March 2021.

Sincerely yours,
On behalf of the MREC Leiden Den Haag Delft,

Ms P.A. Visser
Secretary
