# Supplementary material for: Safety and efficacy of C1-inhibitor in traumatic brain injury (CIAO@TBI): study protocol for a randomized, placebo-controlled, multi-center trial
Source: Trials. 2021 Dec 4;22:874. doi: 10.1186/s13063-021-05833-1 (PMC8642972; doi:10.1186/s13063-021-05833-1)
Supplement: Supplementary file 4 — Additional file 4. Informed consent form. [file 13063_2021_5833_MOESM4_ESM.pdf]

Informatie voor vertegenwoordigers. Proefpersoneninformatie voor deelname aan medisch-wetenschappelijk onderzoek.

CIAO@TBI: Complement Inhibitie: Aanval op Ontsteking na Traumatisch Hersenletsel
Gerandomiseerde evaluatie van Cinryze versus placebo in patiënten met traumatisch hersenletsel.

Inleiding

Geachte heer/mevrouw,

Wij vragen u, als vertegenwoordiger, vriendelijk om goedkeuring te verlenen voor deelname aan wetenschappelijk onderzoek voor uw familielid of naaste (hierna proefpersoon). Meedoen is vrijwillig. Om mee te doen is echter wel uw schriftelijke toestemming nodig. U ontvangt deze brief omdat de proefpersoon een traumatisch hersenletsel heeft doorgemaakt. Voordat u beslist of u toestemming geeft voor deelname van de proefpersoon aan dit onderzoek, krijgt u uitleg over wat het onderzoek inhoudt. Lees deze informatie rustig door en vraag de onderzoeker uitleg als u vragen heeft. U kunt ook de onafhankelijk deskundigen, die aan het eind van deze brief genoemd worden, om aanvullende informatie vragen. U kunt er ook over praten met derden. Zodra de proefpersoon daartoe in staat is, zal deze gevraagd worden in te stemmen met deelname aan het onderzoek. Een beslissing om niet mee te doen heeft geen invloed op de behandeling.
Algemene informatie over meedoen aan zo'n onderzoek vindt u op de website van de Rijksoverheid: www.rijksoverheid.nl/mensenonderzoek.

1. Algemene informatie

Dit onderzoek is opgezet door onderzoeks-neurochirurgen in Leiden, Den Haag, Rotterdam en Amsterdam. Het coördinerende centrum is het Leids Universitair Medisch Centrum (LUMC) en de uitvoering wordt verricht door artsen, onderzoeksverpleegkundigen en onderzoekers in verschillende ziekenhuizen in Nederland. Voor dit onderzoek hebben we de gegevens van 106 proefpersonen nodig. De medisch-ethische toetsingscommissie heeft dit onderzoek goedgekeurd.

2. Doel van het onderzoek

Traumatisch hersenletsel is een belangrijke, maar slechts beperkt begrepen aandoening die jaarlijks meer dan 2.5 miljoen mensen treft in Europa Patiënten die hoofdletsel oplopen

ontwikkelen verhoogde druk in het hoofd door ontsteking. Deze ontsteking kan mogelijk worden verlaagd door het geneesmiddel “Cinryze”. Het doel van dit onderzoek is uitzoeken hoe veilig en werkzaam Cinryze is voor de behandeling van ontsteking bij traumatisch hersenletsel. Cinryze kan nu nog niet worden voorgeschreven door neurochirurgen voor traumatisch hersenletsel.

De werking van Cinryze vergelijken we met de werking van een placebo. Een placebo is een middel zonder werkzame stof, een ‘nepmiddel’.

3. Achtergrond van het onderzoek

Door een ontstekingsreactie na traumatisch hersenletsel ontstaat een zwelling van de hersenen. Door deze zwelling wordt de druk in de schedel te groot, wat kan leiden tot verdere schade aan de hersenen. Dit komt doordat er niet genoeg ruimte voor de hersenen in de schedel is. Uit onderzoek blijkt dat één van de delen van het immuunsysteem, een lichaamssysteem wat bacteriën tegengaat, een grote rol speelt bij het ontstaan van deze zwelling. Met dit onderzoek wordt onderzocht of de ontstekingsreactie door middel van een medicijn dat de reactie remt, zorgt voor minder verdere hersenschade. Het medicijn dat hiervoor gebruikt gaat worden is Cinryze. Momenteel wordt Cinryze nog niet gegeven aan patiënten met traumatisch hersenletsel. Door middel van deze studie wordt gekeken of Cinryze het immuunsysteem genoeg onderdrukt.

4. Wat meedoen inhoudt

Het onderzoek, inclusief de vervolgbezoeken na de behandeling duurt maximaal tot 12 maanden na ontslag uit het ziekenhuis.

Behandeling

We behandelen de proefpersoon bij opname volgens onze normale standaard zorg. Als de proefpersoon meedoet aan het onderzoek, krijgt deze één keer een onderzoeksmiddel toegediend met een infuus in de ader. De helft van alle proefpersonen krijgt behandeling met Cinryze (groep 1) en de andere helft krijgt een placebo toegediend (groep 2). Loting bepaalt welke medicatie de proefpersoon krijgt. De proefpersoon en de onderzoeker weten niet in welke groep de proefpersoon zit. Als het voor de gezondheid van de proefpersoon belangrijk is, kan dit wel worden opgezocht.

Bezoeken en metingen

Tijdens opname zal informatie betreffende de verwondingen en medische voorgeschiedenis verzameld worden. We zullen ook kopieën van scans en resultaten van bloedtesten, die de

proefpersoon in het kader van de medische behandeling krijgt, verzamelen. Voor deze studie wordt zeven keer vier extra buisjes bloed afgenomen om de ontstekingsreactie en de concentratie van het medicijn in het bloed te meten. Waar mogelijk zal het bloed worden afgenomen uit de lijn in de ader die al in het kader van de normale medische behandeling werden geplaatst. Als de proefpersoon in het kader van standaard zorg een drain geplaatst heeft gekregen, zullen we op vijf momenten een buisje hersenvocht afnemen (2 ml). Dit wordt gebruikt om de ontstekingsreactie en de concentratie van het medicijn in het hersenvocht te meten.

In het eerste jaar na de behandeling van de proefpersoon, zal deze 3 keer per post of telefonisch vragenlijsten ontvangen die moet worden ingevuld met betrekking tot hoe het met de proefpersoon gaat. Een telefoongesprek of het invullen van de vragenlijsten kost ongeveer 15 tot 30 minuten. Er zal, indien van toepassing, een gefrankeerde envelop worden meegestuurd zodat de ingevulde vragenlijsten kunt terugsturen naar het postadres in Leiden. In bijlage C staat een overzicht van alle contactmomenten.

Welzijn van de proefpersoon is van hoogste prioriteit in dit onderzoek. Mochten we door klinische of logistieke redenen ervaren dat een onderdeel van het onderzoek niet aangewezen is voor de medische situatie, zullen we dat gedeelte van het onderzoek niet uitvoeren.

5. Wat wordt er van de proefpersoon verwacht bij deelname aan de studie

Om het onderzoek goed te laten verlopen, en voor veiligheid van de proefpersoon, is het belangrijk dat deze zich aan de volgende afspraken houdt.

De afspraken zijn dat de proefpersoon:

- De vragenlijsten naar waarheid invult
- Niet ook nog aan een ander medisch-wetenschappelijk onderzoek meedoet

Het is belangrijk dat de proefpersoon contact opneemt met de onderzoeker:

- Als deze plotseling gezondheidsklachten krijgt of wordt opgenomen/behandeld in een ziekenhuis
- Als deze niet meer wilt meedoen aan het onderzoek.
- Als de contactgegevens wijzigen.

6. Mogelijke risico's van deelname aan de studie

Zoals elk geneesmiddel kan ook Cinryze bijwerkingen hebben, al krijgt niet iedereen daarmee te maken. Dit kunnen onder meer allergie-achtige reacties zijn.

Vaak voorkomende bijwerkingen (kunnen bij maximaal 1 op de 10 mensen optreden):

- *Huiduitslag*

Soms voorkomende bijwerkingen (kunnen bij maximaal 1 op de 100 mensen optreden):

- *Hoge bloedsuiker*
- *Duizeligheid, hoofdpijn*
- *Trombose, pijnlijke aderen, opvliegers*
- *Hoesten*
- *Misselijkheid, braken, diarree, buikpijn*
- *Huidschilfers, jeuk of roodheid*
- *Gewrichtszwelling- en pijn, spierpijn*
- *Uitslag of pijn op de injectieplek*
- *Borstklachten en koorts*

Bijna alle bijwerkingen zijn kortdurend, en zullen zonder verdere behandeling snel overgaan. De proefpersoon moet onmiddellijk contact opnemen met de onderzoeker als deze last krijgt van plotseling piepend ademhalen, ademhalingsproblemen, zwelling van oogleden, gezicht, lippen of onderbenen (met rood/blauwe verkleuring). Voor deze bijwerkingen is goede controle en een mogelijke behandeling noodzakelijk om blijvende klachten te voorkomen.

Het onderzoeksmiddel kan ook bijwerkingen hebben die nog onbekend zijn.
Een bijsluiter van zowel het middel als het placebo middel wordt meegegeven om door te lezen en een weloverwogen beslissing te kunnen maken over deelname aan de studie.

Metingen

Bloedafnames kunnen pijn doen of een bloeduitstorting geven. Per dag nemen we maximaal 15 ml bloed bij de proefpersoon af. Deze hoeveelheid geeft bij volwassenen geen problemen. Om het ongemak zo beperkt mogelijk te houden, wordt waar mogelijk bloed afgenomen uit reeds geplaatste lijnen in de ader. Indien bloed wordt afgenomen via de reeds ingebrachte lijnen, is er geen extra arm prik nodig.
De afname van hersenvocht wordt alleen uitgevoerd als de proefpersoon al een drain geplaatst heeft. Deze afname zal dan geen ongemakken veroorzaken.

Zwangerschap van u of uw partner

Vrouwen die zwanger zijn of borstvoeding geven, kunnen niet meedoen aan dit onderzoek. Er is beperkte informatie over de veiligheid van het gebruik van Cinryze tijdens zwangerschap en borstvoeding.

7. Mogelijke voor- en nadelen

Het is belangrijk dat de mogelijke voor- en nadelen goed worden afgewogen voordat er een besluit wordt gemaakt over meedoen.

Voordelen

Deelname aan deze studie draagt voornamelijk bij aan uitgebreidere kennis en betere medische zorg voor toekomstige patiënten met traumatisch hersenletsel. Voor de proefpersoon betekent deelname aan dit onderzoek niet meteen dat zijn/haar uitkomst beter zal zijn dat wanneer de proefpersoon niet meedoet. Het onderzoeksmiddel kan mogelijk zorgen voor een verlaging van de zwelling en de ontstekingsreactie in het hoofd met goede gevolgen van dien, maar zeker is dat niet. De proefpersoon zal geïnformeerd worden over de onderzoeksresultaten indien deze beschikbaar zijn en deze resultaten zullen worden gedeeld met het klinische team dat verantwoordelijk is voor uw medische behandeling.

Nadelen

Nadelen van meedoen aan het onderzoek kunnen zijn:

- Mogelijke bijwerkingen van Cinryze
- Mogelijke ongemakken van de metingen in het onderzoek, zoals door afname van bloed.

Deelname aan het onderzoek betekent ook:

- dat de proefpersoon extra tijd kwijt is door extra vervolgonderzoeken (tot maximaal 30 minuten per contactmoment; zie punt 4 en bijlage C voor specificaties);
- extra testen en vragenlijsten met mogelijk soms confronterende vragen

Al deze zaken zijn hiervoor onder punt 4, 5 en 6 beschreven.

8. Als u niet wilt meedoen of wilt stoppen met het onderzoek

U beslist voor de proefpersoon of die meedoet aan het onderzoek. Deelname is vrijwillig. Als u niet wilt dat de proefpersoon meedoen, wordt deze op de gebruikelijke manier behandeld.

Als u toestemming geeft, kunnen zowel u als de proefpersoon zich altijd bedenken en alsnog stoppen, ook tijdens het onderzoek. Dit zal de behandeling van de proefpersoon op geen

enkele manier beïnvloeden. Er hoeft niet gezegd te worden waarom er wordt gestopt. Wel moet dit direct gemeld worden aan de onderzoeker.

Tevens is de Code Verzet van toepassing. Dit houdt in dat, wanneer de proefpersoon niet in staat is aan te geven studiedeelname te willen beëindigen (dus wilsonbekwaam), studiedeelname zal worden beëindigd mochten er tekenen van verzet zijn. Hieronder valt onder andere, maar niet uitsluitend: actief protesteren, tekenen van pijn of discomfort of het weigeren van het geven van antwoord. Als er nieuwe informatie over het onderzoek is die belangrijk is voor de proefpersoon, laat de onderzoeker dit weten. Er wordt dan opnieuw aan u gevraagd of de proefpersoon deel blijft nemen.

9. Einde van het onderzoek

Deelname aan het onderzoek stopt als

- alle contactmomenten zoals beschreven onder punt 4 en bijlage C voorbij zijn
- proefpersoon of vertegenwoordig kiest om te stoppen
- de onderzoeker het beter voor de proefpersoon vindt om te stoppen
- het LUMC, de overheid of de beoordelende medisch-ethische toetsingscommissie, besluit om het onderzoek te stoppen.

Het hele onderzoek is afgelopen als de contactmomenten van alle deelnemers zijn afgerond. De medicatie die de proefpersoon gebruikt heeft bij het onderzoek, is niet beschikbaar na afloop van het onderzoek. De onderzoeker kan met de proefpersoon praten over de mogelijkheden voor verdere medische zorg.

Na het verwerken van alle gegevens informeert de onderzoeker de proefpersoon over de belangrijkste uitkomsten van het onderzoek. De onderzoeker kan de proefpersoon dan ook vertellen in welke groep deze zat. Als de proefpersoon dit niet wilt, dan kan deze dit tegen de onderzoeker zeggen. Hij/zij mag het de proefpersoon dan niet vertellen.

10. Gebruik en bewaren van gegevens en lichaamsmateriaal

Voor dit onderzoek worden persoonsgegevens en lichaamsmateriaal verzameld, gebruikt en bewaard. Het gaat om gegevens zoals naam, adres, geboortedatum en om gegevens over gezondheid. Voor dit onderzoek zijn buisjes met bloed nodig. Het verzamelen, gebruiken en bewaren van gegevens en lichaamsmateriaal is nodig om de vragen die in dit onderzoek worden gesteld te kunnen beantwoorden en de resultaten te kunnen publiceren. Wij vragen voor het gebruik van deze gegevens en lichaamsmateriaal toestemming.

Vertrouwelijkheid van gegevens en lichaamsmateriaal
Om de privacy te beschermen krijgen de gegevens en lichaamsmateriaal een elektronische code. Naam en andere gegevens die de proefpersoon direct kunnen identificeren worden los van de onderzoeksgegevens bewaard in het registratiesysteem. Alleen met de sleutel van de code zijn onderzoeksgegevens tot de proefpersoon te herleiden. De gegevens en het lichaamsmateriaal die onder betrokken onderzoekers worden gedeeld bevatten alleen de code, maar geen naam of andere gegevens waarmee identificatie mogelijk wordt. Ook in rapporten en publicaties over het onderzoek zijn de gegevens niet tot de proefpersoon te herleiden.

Een kopie van het getekende toestemmingsformulier zal worden opgeslagen in het LUMC. Ook is het voor dit onderzoek nodig dat de onderzoeker in het LUMC inzage heeft in de persoonsgegevens van de proefpersoon. Hiervoor geeft u apart toestemming op het toestemmingsformulier. In rapporten en publicaties over het onderzoek zijn de gegevens niet tot de proefpersoon te herleiden.

Toegang tot gegevens voor controle
Sommige personen kunnen op de onderzoekslocatie toegang krijgen tot alle gegevens. Ook tot de gegevens zonder code. Dit is nodig om te kunnen controleren of het onderzoek goed en betrouwbaar is uitgevoerd. Personen die ter controle inzage krijgen in deze gegevens zijn: de commissie die de veiligheid van het onderzoek in de gaten houdt, een controleur/monitor die voor het LUMC werkt of die door het LUMC is ingehuurd, de medisch ethische toetsingscommissie die het onderzoek beoordeelde en goedkeurde en nationale toezichthoudende autoriteiten, bijvoorbeeld, de Inspectie Gezondheidszorg en Jeugd. Zij houden de gegevens geheim. Wij vragen ook voor deze inzage toestemming te geven.

Bewaartermijn gegevens en lichaamsmateriaal
Persoonsgegevens moeten 25 jaar worden bewaard in het LUMC. Dit zal gecodeerd gebeuren in een beveiligde data-archief volgens goede onderzoekspraktijken in het lokale ziekenhuis.

Lichaamsmateriaal wordt niet onmiddellijk na gebruik vernietigd. Het wordt bewaard om daarop in de loop van dit onderzoek nog nieuwe bepalingen te kunnen doen die te maken hebben met dit onderzoek. Uw lichaamsmateriaal wordt gecodeerd centraal opgeslagen in het Laboratorium voor Diagnostische Genoomanalyse (LDGA) van het LUMC.

Bewaren en gebruik van gegevens en lichaamsmateriaal voor ander onderzoek

Gegevens en lichaamsmateriaal kunnen na afloop van dit onderzoek ook nog van belang zijn voor ander wetenschappelijk onderzoek op het gebied van behandeling van traumatisch hersenletsel. Daarvoor zullen de gegevens en het lichaamsmateriaal 25 jaar worden bewaard. Gebruik van de gegevens voor toekomstig onderzoek zal enkel worden toegestaan indien het aan de standaarden voldoet wat betreft gegevensbescherming en vertrouwelijkheid zoals beschreven binnen de Europese Unie. Met instemming om deel te nemen aan deze studie geeft u ook toestemming dat data van de proefpersoon uit deze studie gebruikt kan worden door de subsidieverstrekkers of soortgelijke openbare gezondheidsinstituten in Europa voor verdere analyses, bijvoorbeeld om vast te stellen of een van de onderzochte behandelingen een meerwaarde heeft. De onderzoekers die de aanvullende analyses uitvoeren zullen in geen geval de identiteit van de proefpersoon te zien krijgen en alle onderzoekers zijn gebonden aan de geheimhoudingsplicht. U kunt op het toestemmingsformulier aangeven of u hier wel of niet mee instemt. Indien u hier niet mee instemt, kan de proefpersoon gewoon deelnemen aan het huidige onderzoek.

Informatie over onverwachte bevindingen
Tijdens dit onderzoek kan er bij toeval iets gevonden worden dat niet van belang is voor het onderzoek maar wel voor de proefpersoon. Als dit belangrijk is voor de gezondheid, zal de behandelend arts de vertegenwoordiger of de proefpersoon op de hoogte brengen. Dan kan besproken worden wat er moet worden gedaan. Ook hiervoor wordt toestemming gevraagd.

Intrekken toestemming
Toestemming voor gebruik van persoonsgegevens en lichaamsmateriaal kan altijd worden ingetrokken. Dit geldt voor dit onderzoek en ook voor het bewaren en het gebruik voor het toekomstige onderzoek. De onderzoeksgegevens die zijn verzameld tot het moment dat toestemming wordt ingetrokken worden nog wel gebruikt in het onderzoek. Lichaamsmateriaal wordt na intrekking van de toestemming vernietigd. Als er al metingen met dat lichaamsmateriaal zijn gedaan, dan worden die gegevens nog wel gebruikt.

Meer informatie over uw rechten bij verwerking van gegevens
Voor algemene informatie over de rechten bij verwerking van persoonsgegevens kan de website van de Autoriteit Persoonsgegevens worden geraadpleegd.

Bij vragen over rechten kan ook contact worden opgenomen met de verantwoordelijke voor de verwerking van uw persoonsgegevens. . Voor dit onderzoek is dat:

Leids Universitair Medisch Centrum (LUMC). Zie bijlage A voor contactgegevens en website.

Bij vragen of klachten over de verwerking van persoonsgegevens raden we aan eerst contact op te nemen met de onderzoekslocatie.
Bekijk voor meer informatie over privacy het privacy statement van het LUMC op de website van het LUMC: zie bijlage A. U kunt ook contact opnemen met de Functionaris voor de Gegevensbescherming van het LUMC via email: infoavg@lumc.nl of de Autoriteit Persoonsgegevens.

Registratie van het onderzoek
Informatie over dit onderzoek is ook opgenomen in een overzicht van medisch-wetenschappelijke onderzoeken namelijk (https://www.ccmo.nl/over-de-ccmo/websites-van-de-ccmo/ccmo-register). Daarin zijn geen gegevens opgenomen die naar de proefpersoon herleidbaar zijn. Na het onderzoek kan de website een samenvatting van de resultaten van dit onderzoek tonen. U vindt dit onderzoek onder CIAO@TBI.

11. Verzekering voor proefpersonen

Voor iedereen die meedoet aan dit onderzoek is een verzekering afgesloten. De verzekering dekt schade door het onderzoek. Niet alle schade is gedekt. In **bijlage B** vindt u meer informatie over de verzekering en de uitzonderingen. Daar staat ook aan wie u schade kunt melden.

12. Informeren huisarts en/of neurochirurg/apotheker

Wij sturen de huisarts of de neurochirurg/apotheker altijd een brief om te laten weten dat de proefpersoon meedoet aan het onderzoek. Dit is voor eigen veiligheid. Als u dit niet goed vindt, kan de proefpersoon niet meedoen aan dit onderzoek. Voor het versturen van de vragenlijsten kunnen we contact opnemen met de huisarts of de neurochirurg, bijvoorbeeld over de adresgegevens van de proefpersoon.

13. Vergoeding voor meedoen

De deelname aan het onderzoek kost niets. Er wordt daarom ook niet betaald voor deelname aan dit onderzoek.

14. Heeft u vragen?

Bij vragen kunt u contact opnemen met het onderzoeksteam.
Voor onafhankelijk advies over meedoen aan dit onderzoek kunt u ook terecht bij de onafhankelijke arts. Zij weet veel over het onderzoek, maar hebben niets te maken met dit onderzoek.

Indien u of de proefpersoon klachten heeft over het onderzoek, kunt u dit het best bespreken met de onderzoeker of uw behandelend arts. Wilt u dit liever niet, dan kunt u zich beiden wenden tot de klachtencommissie. Alle contactgegevens gegevens zijn te vinden in **bijlage A**: Contactgegevens.

15. Ondertekening toestemmingsformulier

Wanneer u voldoende bedenktijd heeft gehad, wordt u gevraagd te beslissen over deelname aan dit onderzoek. Aangezien toediening van medicatie moet gebeuren binnen 12 uur na het trauma, moet binnen dit termijn de keuze worden gemaakt. Indien u toestemming geeft, zullen wij u vragen deze op de bijbehorende toestemmingsverklaring schriftelijk te bevestigen. Door uw schriftelijke toestemming geeft u aan dat u de informatie heeft begrepen en instemt met deelname aan het onderzoek voor de proefpersoon.

Zowel uzelf als de onderzoeker ontvangen een getekende versie van deze toestemmingsverklaring.

Dank voor uw aandacht.

16. Bijlagen bij deze informatie

A. Contactgegevens voor LUMC

B. Informatie over de verzekering

C. Schema onderzoek handelingen / omschrijving onderzoek handelingen

E. Toestemmingsformulier vertegenwoordiger

Los meegeven:

F. Bijsluiter Cinryze

G. Bijsluiter Placebo

17. Bijlage A: Contactgegevens voor LUMC

Voor aanvullende informatie omtrent de CIAO@TBI trial, gelieve contact op te nemen met:
-I.A.M. van Erp, PhD-student Neurochirurgie
Tel.: 06 11757805, e-mail: I.A.M.van_Erp@lumc.nl

-Researchverpleegkundigen SAINTS Leiden – The Hague
Tel:. 071-5262144, e-mail: TIPS@lumc.nl

Wilt u graag advies over meedoen aan dit onderzoek? Dan kunt u terecht bij een onafhankelijke arts, die niet bij het onderzoek betrokken is:
Dr. Marike Broekman, neurochirurg
E-mail: m.broekman@haaglandenmc.nl

Bij klachten kunt u zich melden bij het patiëntenservicebureau in het LUMC, locatie H2-11 (routenummer 473, tegenover het Leidseplein). Hier kunt u melding maken van uw onvrede en het klachtenformulier invullen. Het patiëntenservicebureau informeert u zo snel mogelijk over een mogelijke oplossing en kan eventueel de klachtenfunctionaris inschakelen. U kunt ook het klachtenformulier digitaal invullen. Zie website LUMC pagina klacht indienen

Wilt u contact opnemen met de klachtenfunctionaris of de functionaris van de gegevensbescherming van het LUMC?
-Klachten: Patiëntenservicebureau, locatie H2-11 (routenummer 473)
Tel.: 071 5262989, e-mail: patientenservicebureau@lumc.nl
-Functionaris voor de gegevensbescherming van de instelling:
e-mail: infoavg@lumc.nl

Voor meer informatie over uw rechten: https://www.lumc.nl/12367

LEIDS UNIVERSITAIR MEDISCH CENTRUM

Bijlage B: informatie over de verzekering

Voor iedereen die meedoet aan dit onderzoek, heeft het Leids Universitair Medisch Centrum een verzekering afgesloten. De verzekering dekt schade door deelname aan het onderzoek. Dit geldt voor schade tijdens het onderzoek of binnen vier jaar na het einde van uw deelname aan het onderzoek. Schade moet u binnen die vier jaar aan de verzekeraar hebben gemeld.

De verzekering dekt niet alle schade. Onderaan deze tekst staat in het kort welke schade niet wordt gedekt.
Deze bepalingen staan in het 'Besluit verplichte verzekering bij medisch-wetenschappelijk onderzoek met mensen 2015'. Dit besluit staat in de Wettenbank van de overheid (https://wetten.overheid.nl).

Bij schade kunt u direct contact leggen met de verzekeraar [of schaderegelaar].

De verzekeraar van het onderzoek is:	
Naam:	Centramed B.A.
Adres:	Maria Montessorilaan 9, 2719 DB Zoetermeer, Nederland
Telefoonnummer:	+31 703017070
E-mail:	info@centramed.nl

De verzekering biedt een dekking van € 650.000 per proefpersoon en € 5.000.000 voor het hele onderzoek € 7.500.000 per jaar voor alle onderzoeken van dezelfde opdrachtgever.

De verzekering dekt de volgende schade **niet**:

- schade door een risico waarover u in de schriftelijke informatie bent ingelicht. Dit geldt niet als het risico zich ernstiger voordoet dan was voorzien of als het risico heel onwaarschijnlijk was;
- schade aan uw gezondheid die ook zou zijn ontstaan als u niet aan het onderzoek had meegedaan;
- schade door het niet (volledig) opvolgen van aanwijzingen of instructies;
- schade aan uw nakomelingen, als gevolg van een negatief effect van het onderzoek op u of uw nakomelingen;
- schade door een bestaande behandelmethode bij onderzoek naar bestaande behandelmethoden.

Bijlage C – Overzicht metingen

Tijdens opname:

TIJD	HANDELING
Na geven van toestemming	Afname 4 bloedbuizen (max. 15 ML)
Na afname buis bloed	CINRYZE of placebo toediening
6 uur na medicatie toediening	Afname 4 bloedbuizen (max. 15 ML)
12 uur na medicatie toediening	Afname 4 bloedbuizen (max. 15 ML)
24 uur na medicatie toediening	Afname 4 bloedbuizen (max. 15 ML)
48 uur na medicatie toediening	Afname 4 bloedbuizen (max. 15 ML)
72 uur na medicatie toediening	Afname 4 bloedbuizen (max. 15 ML)
96 uur na medicatie toediening	Afname 4 bloedbuizen (max. 15 ML)

ENKEL indien een drain is geplaatst voor standaard zorg:

TIJD	HANDELING
Na geven van toestemming	Afname 1 buis (2ml)
Na afname buis bloed	CINRYZE of placebo toediening
24 uur na medicatie toediening	Afname 1 buis (2ml)
48 uur na medicatie toediening	Afname 1 buis (2ml)
72 uur na medicatie toediening	Afname 1 buis (2ml)
96 uur na medicatie toediening	Afname 1 buis (2ml)

Na ontslag uit ziekenhuis telefonisch/per post:

TIJD	HANDELING
Ontslag	Afname van 1 vragenlijst (max. 10 min.)
3 maanden na ontslag	Afname van 4 vragenlijsten (max. 15 min.)
6 maanden na ontslag	Afname van 6 vragenlijsten (max. 30 min.)
12 maanden na ontslag	Afname van 6 vragenlijsten (max. 30 min.)

LEIDS UNIVERSITAIR MEDISCH CENTRUM

Bijlage D: Toestemmingsformulier vertegenwoordiger

CIAO@TBI: Complement inhibitie als behandeling van traumatisch hersenletsel

Ik ben gevraagd om toestemming te geven voor deelname van de volgende persoon aan dit medisch-wetenschappelijke onderzoek:

Naam proefpersoon: Geboortedatum: __ / __ / __

- Ik heb de informatiebrief voor de proefpersoon/vertegenwoordiger gelezen. Ook kon ik vragen stellen. Mijn vragen zijn voldoende beantwoord. Ik had genoeg tijd om te beslissen of ik wil dat deze persoon meedoet.
- Ik weet dat meedoen vrijwillig is. Ook weet ik dat ik op ieder moment kan beslissen dat deze persoon toch niet mee doet. Daarvoor hoef ik geen reden te geven.
- Ik geef toestemming voor het informeren van de huisarts/neurochirurg/apotheker van de proefpersoon dat hij/zij meedoe aan dit onderzoek.
- Ik geef toestemming om, indien nodig, informatie op te vragen bij de huisarts/neurochirurg/apotheker van de proefpersoon over mogelijke complicaties en over adresgegevens
- Ik geef toestemming voor het verzamelen en gebruiken van de gegevens en lichaamsmateriaal van deze persoon voor de beantwoording van de onderzoeksvraag in dit onderzoek en voor de registratie van het onderzoekgeneesmiddel in traumatisch hersenletsel patiënten.
- Ik geef toestemming voor het opslaan van een (kopie) van het getekende toestemmingsformulier in het LUMC.
- Ik weet dat voor de controle van het onderzoek sommige mensen toegang tot alle gegevens van deze persoon kunnen krijgen. Die mensen staan vermeld in deze informatiebrief. Ik geef toestemming voor die inzage door deze personen.
- Ik geef toestemming voor het informeren van de huisarts en/of behandelend specialist van onverwachte bevindingen die van belang (kunnen) zijn voor de gezondheid van de proefpersoon.
- Ik weet dat deze persoon, op het moment dat die zelf kan beslissen, toestemming gevraagd zal worden voor het voortzetten van de studie.
- Ik geef ☐ **wel**

□ **geen**
toestemming om de persoonsgegevens van deze persoon langer te bewaren en te gebruiken voor toekomstig onderzoek op het gebied van traumatisch hersenletsel.

- Ik geef □ **wel**

  □ **geen**
  toestemming om het lichaamsmateriaal na dit onderzoek te bewaren en om dit later nog voor meer en ander onderzoek te gebruiken, zoals in de informatiebrief staat.

- Ik geef □ **wel**

  □ **geen**
  toestemming om deze persoon na dit onderzoek opnieuw te benaderen voor een vervolgonderzoek.

- Ik wil □ **wel**

  □ **niet**
  dat de proefpersoon geïnformeerd wordt over welke behandeling deze heeft gehad/in welke groep deze zat.

- Ik geef □ **wel**

  □ **geen**

  toestemming om, in het geval de proefpersoon tijdens de looptijd van het onderzoek zou komen te overlijden, de officiële doodsoorzaakgegevens op te vragen bij het Centraal Bureau voor de Statistiek

- Ik ga ermee akkoord dat deze persoon meedoet aan dit onderzoek.

Naam wettelijk vertegenwoordiger:
Relatie tot de proefpersoon:
Handtekening: Datum: __ / __ / __

Ik verklaar hierbij dat ik deze persoon/personen volledig heb geïnformeerd over het genoemde onderzoek.

Als er tijdens het onderzoek informatie bekend wordt die de toestemming van de wettelijk vertegenwoordiger zou kunnen beïnvloeden, dan breng ik hem/haar daarvan tijdig op de hoogte.

Leids Universitair Medisch Centrum

Naam onderzoeker (of diens vertegenwoordiger):

Handtekening: Datum: __ / __ / __

Aanvullende informatie is gegeven door (indien van toepassing):

Naam:

Functie:

Handtekening: Datum: __ / __ / __

De vertegenwoordiger krijgt een volledige informatiebrief mee, samen met een getekende versie van het toestemmingsformulier.
