# Supplementary material for: Safety and efficacy of C1-inhibitor in traumatic brain injury (CIAO@TBI): study protocol for a randomized, placebo-controlled, multi-center trial
Source: Trials. 2021 Dec 4;22:874. doi: 10.1186/s13063-021-05833-1 (PMC8642972; doi:10.1186/s13063-021-05833-1)
Supplement: Supplementary file 3 — Additional file 3. Copy of the original funding documentation. [file Additional file 3b. Original funding documentation.pdf › Additional file 3b. Original funding documentation.pdf]

Hersenstichting

Prof. dr. Wilco Peul
Leiden University Medical Center
Albinusdreef 2
2333 ZA Leiden, The Netherlands

Den Haag, 21-04-2021

Regarding: Support
Title: CIAO@TBI: Complement Inhibition: Attacking the overshooting neuroinflammation after traumatic brain injury

Dear Prof. Wilco Peul ,

In December 2019 The Brain Foundation Netherlands has reviewed your proposal and preliminary granted you a total sum of €583.893.

We, hereby, are pleased to inform you that you have succesfully fulfilled all the criteria to meet our requirements. Therefore the preliminary funding is converted into a definitive grant for the study titled '*CIAO@TBI: Complement Inhibition: Attacking the overshooting neuroinflammation after traumatic brain injury*'.

The conditions for this project are documented in the subsidy agreement that is signed by both parties.

With kind regards,

Merel Heimens Visser
Director

Geef om je hersen

12 December 2019

Request ID: IIR-NLD-002790

Study Title: CIAO@TBI: Complement Inhibition: Attacking the Overshooting inflammation in @fter TBI

Dear Prof. Wilco Peul,

The Shire IIR Review Committee has carefully reviewed your Investigator-Initiated Research proposal entitled "*CIAO@TBI: Complement Inhibition: Attacking the Overshooting inflammation in @fter TBI.*"

We are pleased to inform you that the Committee has decided that Shire will support your proposal. Any and all support from Shire for your research is conditional upon the execution of a mutually agreeable research agreement with all necessary parties.

We look forward to the results from your study.

Thank you for your interest in Shire.

Sincerely,

On behalf of Scientific Review Committee,

Joanna Bancerek

Joanna Bancerek, PhD

Investigator-Initiated Research Program Lead, Hematology & HAE

Global Medical Affairs

Takeda

Donau-City-Strasse 7, DC Tower 1

1220 Vienna, Austria

T +43 1 20100 247 6834

M +43 664 88 120 475

Joanna.Bancerek@Takeda.com
